# Percutaneously inserted ventriculo-ureteral shunt as a salvage treatment in paediatric hydrocephalus: a technical note

**DOI:** 10.1007/s00381-022-05673-7

**Published:** 2022-09-21

**Authors:** Ulrika Sandvik, Jiri Bartek, Erik Edström, Mattias Jönsson, Jakob Stenman

**Affiliations:** 1 Department of Neurosurgery, Karolinska University Hospital, 171 76 Stockholm, Sweden; 2 Department of Clinical Neuroscience, Karolinska Institutet, Stockholm, Sweden; 3 Department of Neurosurgery, Rigshospitalet, Copenhagen, Denmark; 4 Department of Pediatric Radiology, Karolinska University Hospital, Stockholm, Sweden; 5 Department of Pediatric Surgery, Karolinska University Hospital, Stockholm, Sweden; 6 Department of Women’s and Children’s Health, Karolinska Institutet, Stockholm, Sweden

**Keywords:** Hydrocephalus, Ventriculo-ureteral shunt, Paediatric neurosurgery

## Abstract

**Background:**

Hydrocephalus is a challenge for paediatric neurosurgeons. When the abdominal cavity and heart fail as diversion sites for cerebrospinal fluid (CSF), many of the otherwise used alternative diversion sites are not feasible due to the smaller physical body size of children and infants. Using the urinary system as a site of diversion has been described in adults primarily.

**Objective:**

To describe a minimally invasive procedure to percutaneously access the ureter for placement of a distal catheter in the treatment of paediatric hydrocephalus.

**Methods:**

A percutaneous ultrasound-assisted technique was used to access the renal pelvis for catheter placement into the distal ureter.

**Results:**

Fifteen months after the surgery, the child has a stable neurological condition and adequately managed hydrocephalus.

**Conclusion:**

The urinary tract should be considered a viable option for CSF diversion in complex paediatric hydrocephalus. A multidisciplinary approach consisting of interventional radiologists, urologists and neurosurgeons should be involved in the evaluation of potential candidates.

## Introduction

Complex hydrocephalus is a challenge among prematurely born children with posthemorrhagic hydrocephalus. More than 80% of shunt patients require revision and more than half of the hydrocephalic patients are subjected to more than four surgeries [1]. The standard method of diverting cerebrospinal fluid (CSF) into the abdominal cavity is not always feasible. Sometimes, peritoneal resorptive insufficiency arises making other sites of diversion necessary. Current literature describes up to 36 sites of diversion varying from the mastoid bone to the pleura and the fallopian tubes [1]. Finding a site of diversion in a paediatric patient is challenging due to the small size of the patient and often high drainage volumes. The ventriculo-ureteral (VU) shunt is rarely used by the neurosurgical community and not many cases have been reported. Most of the reported cases are adult individuals and a percutaneous technique has rarely been used. In this paper, we describe a complex paediatric case that was successfully managed by the use of a percutaneously inserted VU shunt.

## Case description

The patient is a premature boy, born in gestation week 26 + 2 with a birth weight of 1068 g. He was treated in neonatal intensive care and suffered from both necrotizing enterocolitis and grade IV intraventricular haemorrhage and posthemorrhagic hydrocephalus. His abdominal condition required two laparotomies with intestinal resections, while his posthemorrhagic condition included 32 shunt procedures/revisions (Table 1). He initially underwent implantation of a ventriculoperitoneal (VP) shunt, which due to infection and peritoneal malabsorption was replaced with a ventriculoatrial (VA) shunt. Due to atrial catheter malfunction, a VP shunt was reimplanted. However, also this VP shunt had to be replaced with another VA shunt, secondary to problems with malabsorption and the development of a CSF cyst that caused right-sided hydronephrosis. Unfortunately, extensive central venous thrombosis with subsequent vena cava syndrome eventually required the removal of the second VA shunt. A pleural shunt was now being considered, but due to the large drainage volumes, this was abandoned. The urinary bladder was also considered, but due to episodes of documented non-symptomatic bacteriuria during the past year, this was believed to be a risky endeavour. Finally, a VU shunt remained a viable option, and while acknowledging the risks of urinary tract infections as well as the risk of the shunt becoming a nidus for the formation of urinary calculi, the VU shunt was deemed the best option for this patient.

The parents have consented to the publication of the case.

## Preoperative work-up

An MRI of the brain was performed to rule out hematoma, subdural collections, or new adhesions. Due to a left-sided posthemorrhagic ventricular dilatation, this side was chosen for the proximal catheter. CSF cultures were performed and found negative. Repeated urinary cultures showed no signs of bacteriuria. Persistent hydronephrosis was ruled out by ultrasound. Abdominal MRI showed that both kidneys were normal in size with adequate parenchymal thickness. The right ureter was found to be slightly wider than the left. A review of a previous CT scan showed a right-sided ureteric dilatation, sufficient to harbour the distal catheter. A voiding cystourethrography (VCUG) was performed to rule out vesicoureteral reflux while the bladder volume was estimated to be approximately 150 ml. A high-pressure bladder was considered unlikely, as there were no signs of bladder trabeculation or diverticulae on VCUG and both ultrasound and MRI showed a thin-walled bladder of normal size. Cystometry was not performed.

## Surgical technique

**Fig. 1:**
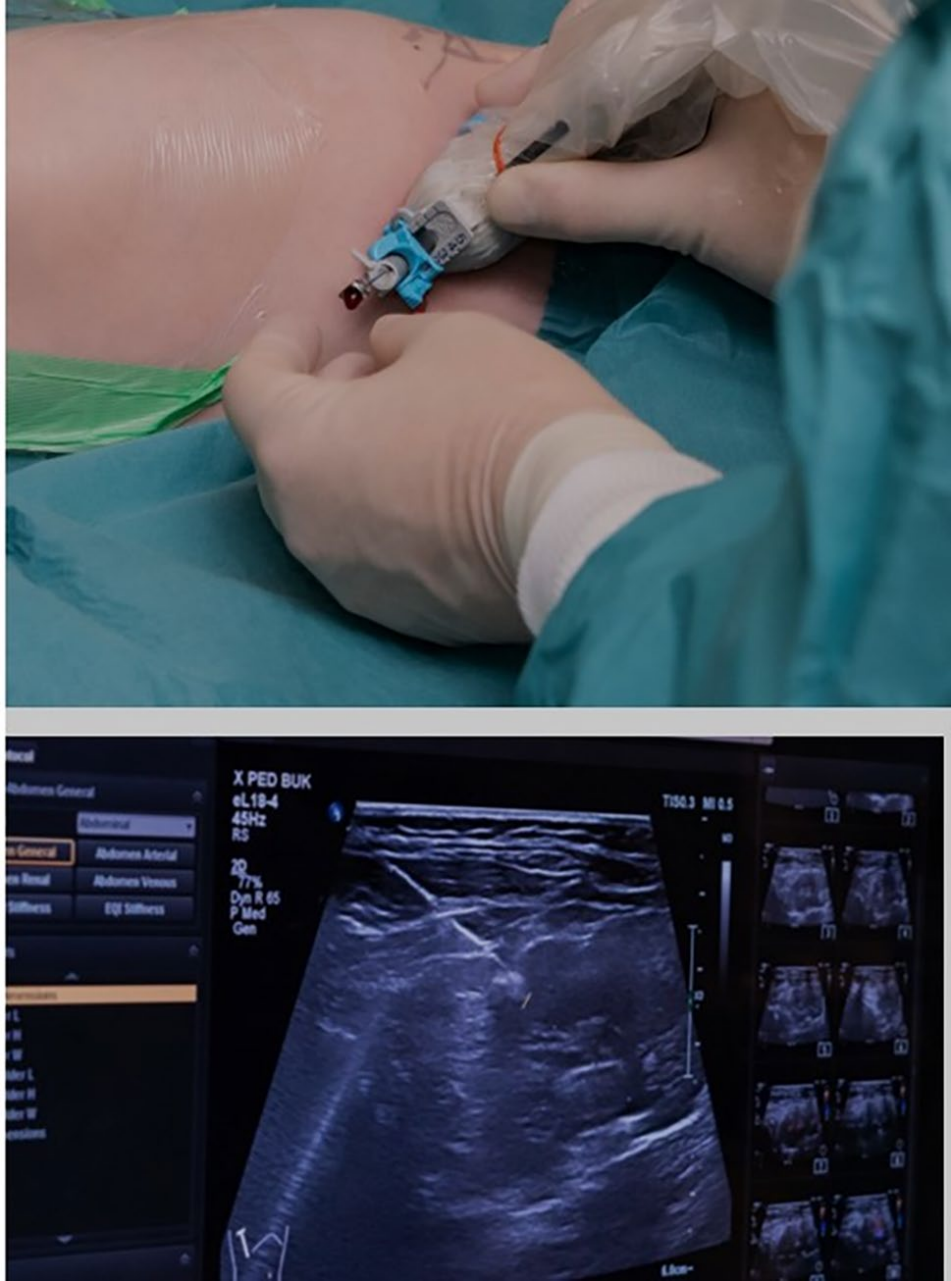
Ultrasound-guided percutaneous insertion of the distal catheter

**Fig. 2:**
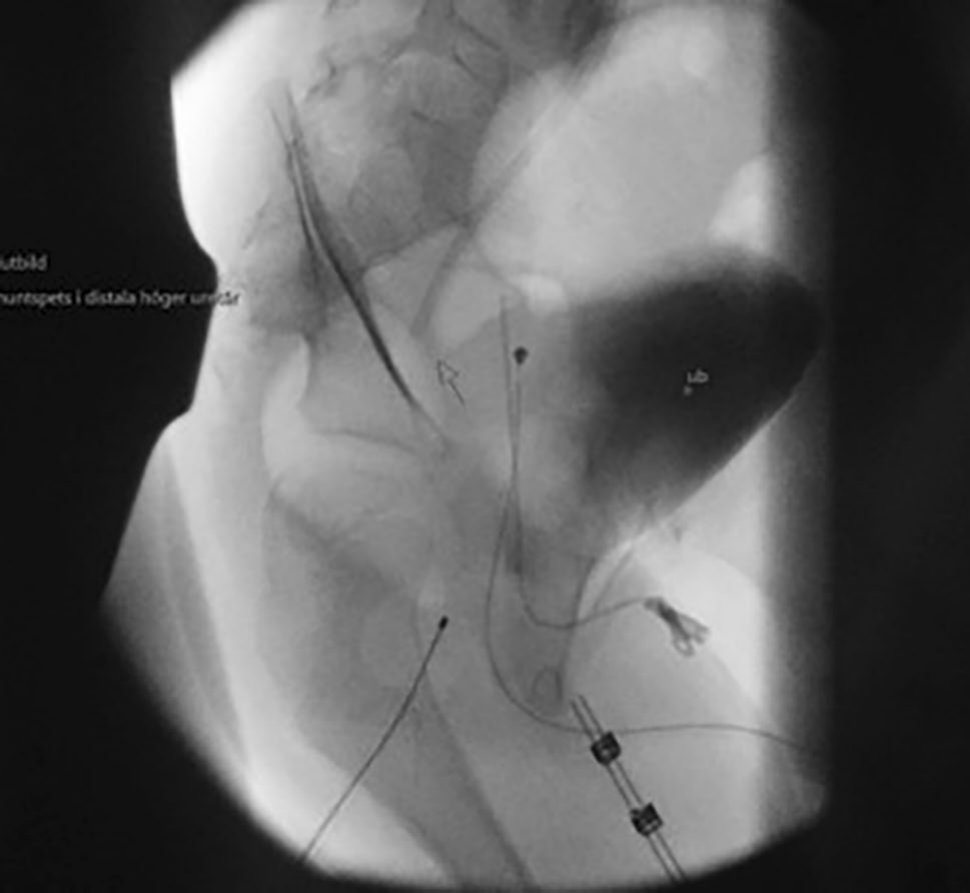
Intraoperative imaging of the distal catheter

**Fig. 3:**
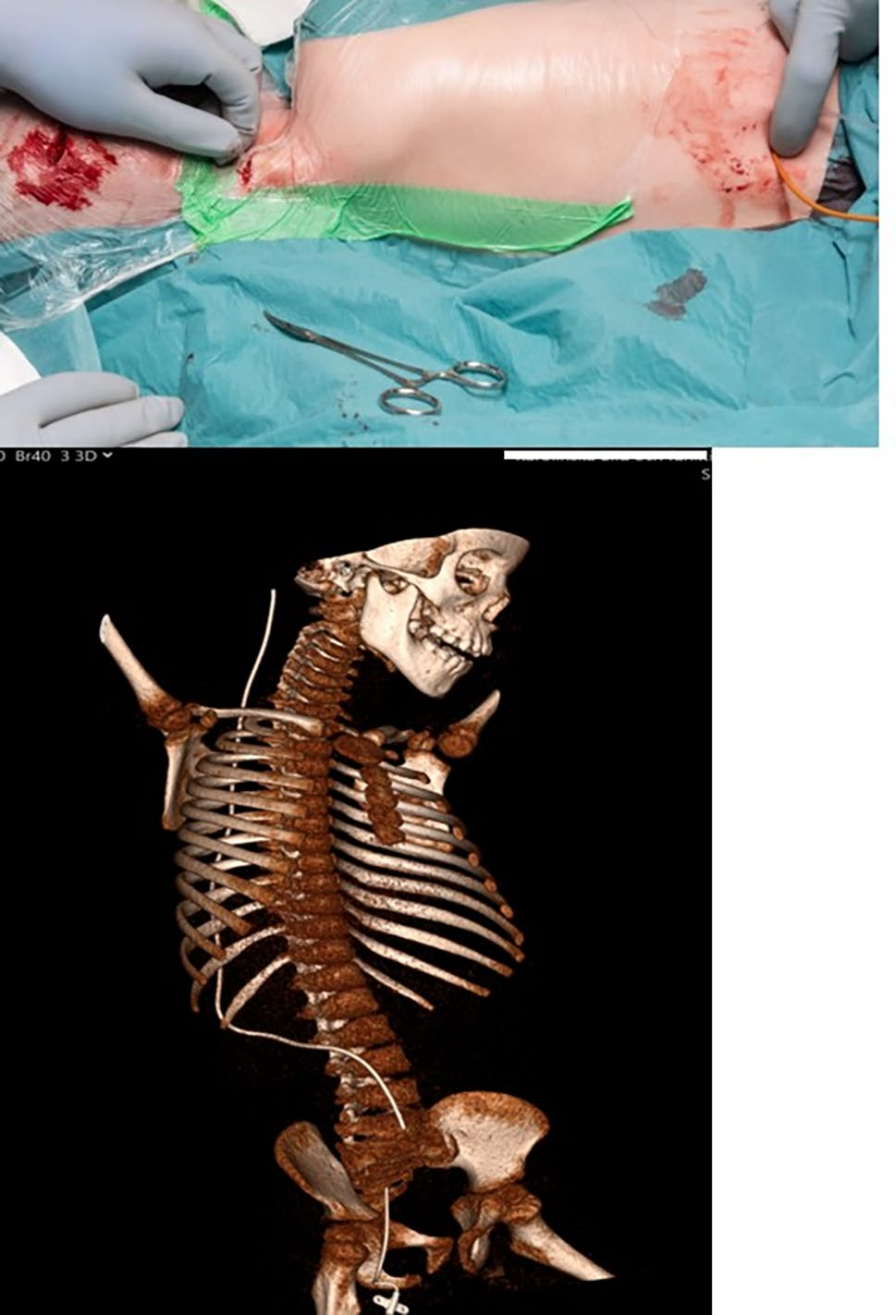
Tunnelling of the shunt dorsally and connection to the valve

The child was positioned semi-prone with his right side slightly elevated. A radiologist experienced in ultrasound-assisted percutaneous nephrostomies (MJ) and a paediatric urologist (JS) were present for the implantation of the distal catheter. The renal pelvis was reached with a micropuncture technique (0.9-mm needle and 0.018-in. guidewire) under ultrasound guidance and fluoroscopic control. Using the Seldinger technique and serial dilatation to 8 F, PTFE-coated stainless steel, 0.035-in. (0.89 mm) guidewire was placed via the ureter into the bladder (Fig. 1). After a small skin incision, the distal shunt catheter was then placed 3 cm cranially to the ureteric orifice and the final position was verified with fluoroscopy (Fig. 2). The shunt catheter was then tunnelled subcutaneously over the back, medially to the right scapula and connected to the shunt valve of a left-sided frontal ventricular catheter (inserted by neurosurgeons JB and US, Fig. 3).

On postoperative day 2, the child developed new neurological symptoms and a CT of the abdominal cavity showed that the distal catheter had migrated 2 cm distally, close to the ureteric orifice where the ureter typically is slightly narrower. There is also the possibility of the tubing being clogged by blood. The problem was resolved by shortening and flushing the distal catheter. The catheter was shortened by 2 cm (neurosurgeon EE). After this revision, the recovery was uneventful.

## Outcome

Follow-up at 6 months included urinary cultures and a low-dose CT to rule out the development of hydronephrosis or urinary calculi, as well as any possible formation of calcifications on the distal catheter. The patient remains on a daily dose of oral prophylactic antibiotics (trimethoprim-sulfamethoxazole). At the 15-month follow-up, there have been no urinary tract or CNS infections, and no further episodes of shunt dysfunction. A new MRI of the brain was performed 1 year postoperatively and showed expected ventricular size and adequate location of the proximal catheter. No new abnormalities have emerged on radiological imaging, apart from those related to the pre-existing condition. There have not been any problems related to the maintenance of a normal electrolyte homeostasis and the patient has not required any form of fluid or electrolyte substitution. Neurological follow-up has consisted of out-patient appointments at the neurosurgical department, initially after 4 weeks and further every 6 months.

## Discussion

Diversion of CSF into the genitourinary system was suggested as early as 1925 when Heile proposed an ureterodural anastomosis by suturing the renal pelvis into the lumbar dura [2]. In 1949, Matson described using a polyethene tube for drainage into the ureter [3]. The idea of utilizing the urinary tract for CSF diversion relies on rapid elimination through micturition rather than absorption [1, 4, 5].

The urinary bladder has long been used as an alternative diversion site in the treatment of hydrocephalus. Development of the VU shunt was initially described as a procedure including nephrectomy and was later developed into a ventriculo-pyelo-ureterostomy, a ventriculorenal shunt, where the distal end of the shunt catheter was placed in the pyelocaliceal area [1, 6]. Since the initial method of implanting a VU-shunt required removal of a kidney, this method never became widely adopted. Later, open surgical techniques sparing the kidney were described [7]. In the 1980s, Smith et al. described a technique of low ureteral transection for distal catheter placement, combined with re-implantation of the ureter, thus avoiding a nephrectomy [7]. The introduction of percutaneous nephrostomy has opened up new possibilities for minimally invasive access to the ureters as a diversion site [8].

Only a few cases of VU shunts have been reported in the literature and several case reports describe adult patients where the size of the ureters is more favourable for this procedure [6, 7, 9–17]. One of the few long-term follow-ups described good results in four patients, provided they had a low-pressure urinary bladder without urinary tract infections [16]. This study included a child, two teenagers, and a 29-year-old man, both of them having an open surgical approach to the ureter. The study describes a 5-year mean survival of the shunt, but all patients eventually needed a re-operation [16]. Complications such as shunt obstruction, infection (with and without associated urinary tract infection), migration or kinking of the tubing, and metabolic complications were described [16]. Many failures have been described to be due to calcification of the distal catheter, which is a known complication of ureteral stenting [9]. There is also the theoretical risk for retrograde reflux of urine into CSF spaces, although this has not been described in any cases [12]. A high-pressure bladder should be ruled out before considering a VU shunt. It also seems that patients with VU shunts might be prone to symptomatic electrolyte imbalance in situations of dehydration or gastroenteritis [3, 12]. Other potential long-term problems in addition to calcifications include biofilm formation on the shunt and erosion of the ureteral wall due to the catheter [12, 18]. Percutaneous insertion, with similar techniques as the one we used, has previously been described by Pillai et al. and Subramanian et al., where two adults with postinfectious and posttraumatic hydrocephalus were successfully treated [8, 12]. To the best of our knowledge, this is the first description of the percutaneous insertion of a VU shunt in a child.

## Conclusion

We have demonstrated the successful use of a percutaneously inserted VU-shunt in a 4-year-old child. The ureters have a high capacity for distension and can accommodate shunts with a larger diameter than those typically recommended for ureteric stenting in children. VU-shunting can thus provide an alternative for salvage CSF drainage even in relatively small children. A multidisciplinary team consisting of neurosurgeons, urologists, and interventional radiologists is essential in evaluating suitable candidates.

**Table 1:** Clinical events and procedures

Age	Clinical event
Born gestational week 26 + 2	Birth weight 1068 g
1 week	Intraventricular hematoma grade IV and development of hydrocephalus
	Sepsis (*Staphylococcus aureus*)
2 weeks, 4 weeks	Two abdominal surgeries due to necrotizing enterocolitis
3 weeks	Intraventricular reservoir for CSF drainage
3 months	Unilateral ventricular dilatation, intracranial endoscopy and bilateral intracranial reservoir
3, 5 months	Left-sided ventriculoperitoneal shunt
4 months	Shunt infection (*Staphylococcus aureus*) and removal of the shunt
4 months	Removal of the right-sided reservoir, ventriculostomy
4 months	Change of ventriculostomy due to catheter occlusion
4,5 months	Left-sided ventriculoperitoneal shunt
5,5 months	Wound revision due to CSF-leakage
6 months	Insufficient peritoneal resorption of CSF, conversion to VA shunt
1 year	Revision distal catheter of VA shunt
1,5 years	Revision distal catheter of VA shunt
2 years	Dysfunction atrial catheter, conversion to VP shunt
2 years 10 months	Overdrainage, valve change from Strata to Certas shunt valve
2 years 10 months	Reoperation due to shunt tube kinking close to the valve
3 years 4 months	Shunt revision due to proximal catheter dysfunction
3 years 4 months	Proximal dysfunction, implantation of a new shunt system
3 years 10 months	Insufficient peritoneal resorption of CSF and development of an abdominal cyst. Low-grade infection with Proprione Acnees
3 years 11 months	Conversion into VA shunt
3 years 11 months	Distal catheter dysfunction due to severe central venous thrombosis and vena cava syndrome. Externalization of the distal catheter
3 years 11 months	Conversion to VP shunt
4 years 4 months	Distal catheter dysfunction due to insufficient peritoneal resorption of CSF, repositioning of the catheter in the abdominal cavity
4 years 4 months	Peritoneal malabsorption, two ultrasound-assisted punctures of the abdomen
4 years 5 months	MRI shows aqueductal obstruction, endoscopic ventriculocisternostomy and aqueductoplasty were performed to evaluate the possibility of shunt-independency
4 years 5 months	Posterior fossa exploration with fenestration of membranes and retrograde endoscopic aqueductoplasty via the fourth ventricle to further increase the chance of shunt independency. Oral acetazolamide treatment started to reduce CSF production. This was discontinued due to hypochloremic metabolic acidosis
4 years 5 months	Increasing abdominal tension due to accumulation of CSF in the abdomen, shunt externalized
4 years 6 months	Endoscopy, re-aqueductoplasty, removal of shunt and insertion of ventriculostomy. Weaning of CSF drainage and child is shunt free one week
4 years 7 months	Leakage of CSF through incisions and insertion of ventriculostomy
4 years 7 months	Insertion of a new VU shunt system
4 years 7 months	Shunt revision with shortening of the distal catheter
5 years 10 months	Stable hydrocephalus, regular follow-ups neurologist, paediatric neurosurgeon and paediatric urologist

## Data Availability

Not applicable.

## Abbreviations

CSF: Cerebrospinal fluid
VU shunt: Ventriculo-ureteral shunt
VP shunt: Ventriuloperitoneal shunt
VCUG: Voiding cystourethrography
F: French scale
